# Mapping the Kitchen Microbiota in Five European Countries Reveals a Set of Core Bacteria across Countries, Kitchen Surfaces, and Cleaning Utensils

**DOI:** 10.1128/aem.00267-23

**Published:** 2023-05-31

**Authors:** Birgitte Moen, Solveig Langsrud, Ingunn Berget, Tove Maugesten, Trond Møretrø

**Affiliations:** a Nofima—Norwegian Institute of Food, Fisheries and Aquaculture Research, Ås, Norway; Centers for Disease Control and Prevention

**Keywords:** microbiome, Illumina, core microbiota, sponges, 16S rRNA gene

## Abstract

The residential kitchen is often heavily colonized by microbes originating from different sources, including food and human contact. Although a few studies have reported the bacterial composition in cleaning utensils and surface samples there is limited knowledge of the bacterial diversity across different sample types, households, and countries. As part of a large European study, we have identified the microbiota of 302 samples from cleaning utensils (sponges and cloths), kitchen surfaces (sinks, cutting boards, countertops, tap handles, and a pooled sample of other handles) in 74 households across 5 countries (France, Hungary, Norway, Portugal, and Romania). In total, 31 bacterial phyla were identified, with *Proteobacteria*, *Firmicutes*, *Bacteroidota*, and *Actinobacteria* being the most abundant. Despite large variations in households with respect to kitchen standards, kitchen practices, cleaning regimes, and diet and considerable differences in bacterial diversity between samples, eight bacterial genera/families commonly associated with environmental sources were identified in most samples and defined as a core microbiota: Acinetobacter, Pseudomonas, *Enhydrobacter*, *Enterobacteriaceae*, *Psychrobacter*, *Chryseobacterium*, *Bacillus*, and Staphylococcus. These genera/families were also among the bacteria with the highest relative abundance across all samples, in addition to *Yersiniaceae*, *Kocuria*, *Pantoea*, and Streptococcus. Taxa associated with potential pathogens and fecal indicators were low in abundance but broadly distributed throughout the households. The microbial composition of surface samples indicated that the microbial composition on kitchen surfaces is more characteristic for the particular country than the object type, while the microbiota of cleaning utensils was similar across countries but differed between types (sponge or cloth).

**IMPORTANCE** There is limited knowledge of the characteristics, differences, and similarities of the bacterial composition in residential kitchens. Here, we report the microbiota of cleaning utensils (sponges and cloths) and five different surface samples in 74 households across five European countries. In addition to increasing the knowledge of the kitchen microbiota from many geographical areas, this study identified a core microbiota in European residential kitchens despite large variations in kitchen practices and kitchen design and standards across countries and households.

## INTRODUCTION

Humans are constantly exposed to a wide range of microorganisms that could impact our health both positively and negatively. Much of the microbial exposure happens indoors, as the indoor microbiome consists of a wide range of microorganisms (1, 2). Within the domestic environment, bacterial colonization has been shown to be highest in kitchens (3–6). Kitchens are readily contaminated with microbes originating from handling and preparation of food, water, air, and humans (7). Also, contamination from kitchen sponges is an important source of bacterial spread in kitchens, as they have been shown to be the most contaminated items in a household (4, 8–10). Kitchen sponges absorb microorganisms, food soils, and humidity during use and dry slowly (11), which leads to growth of bacteria to high levels. Foodborne illness has also been associated with sponges transferring pathogens to food or hands (12–14), and we have previously reported high survival of Salmonella added to used sponges (11). Although previous studies of kitchen sponges have reported dominance of nonpathogenic bacteria, there have been reports of taxonomic relationships to risk group 2 species (moderate individual risk, low community risk) such as Acinetobacter johnsonii, Acinetobacter pittii, Acinetobacter ursingii, Chryseobacterium hominis, and Moraxella osloensis (1, 14). We have previously reported a common set of bacteria in brushes and sponges across two countries (Norway and Portugal) despite the total bacterial levels varying by several magnitudes (12). Many of the same genera also dominated in sponges in two German studies (13, 14). Based on this, we suggested that there may be a set of common bacteria, with Acinetobacter, *Enhydrobacter*, Pseudomonas, and *Chryseobacterium* as the most dominant, in used sponges and brushes across different countries in Europe (12).

Besides the studies on sponges (and brushes), there have been some studies on the kitchen microbiota (2, 7, 15, 16) as well as other indoor environments (1, 2, 16–18). Kitchen surfaces have been shown to be dominated by *Moraxellaceae*, *Streptococcaceae*, *Micrococcaceae*, and *Flavobacteriaceae* (7), and Adams et al. (2) concluded that *Streptophyta*, *Enhydrobacter*, *Mitochondria*, Acinetobacter, and Pseudomonas were closely associated with kitchens. We have previously also shown that *Moraxellaceae* (genus *Moraxella*/*Enhydrobacter*), *Micrococcaceae* (genus *Kocuria*), *Streptococcaceae*, and *Enterobacteriaceae* dominated in sinks from Norwegian households (19). Except for the meta study by Adams et al., (2), who reported that Acinetobacter was abundant in kitchens regardless of geographic location, these studies have been conducted on limited geographical area, making it difficult to identify a common kitchen microbiota or say anything about the diversity between households and cultures. There are indications that the bacterial community on residential surfaces generally reflects the usage pattern (2, 7, 16, 20); e.g., toilets are more similar to other toilets than to other surfaces in restrooms. However, to our knowledge, there are no studies comparing bacterial communities for kitchen samples across several households and countries. Such studies are needed to evaluate if a core microbiota exists in kitchens in general.

The aim of the current study was to map the microbiota in domestic kitchens, evaluate if a kitchen core microbiota exists, and evaluate potential associations of the microbiota with the household country and/or objects. The microbiota was identified in 302 samples from cleaning utensils and kitchen surfaces (including hand contact points) in 74 households across five countries (France, Hungary, Norway, Portugal, and Romania). To our knowledge this is the first published work that reports on the kitchen microbiota across several countries.

## RESULTS

### Sequencing data.

In total, 305 samples were analyzed and, altogether, 3,487 sub-operational taxonomic units (sOTUs; closely related bacterial sequences with single nucleotide differences) were detected from a total of 18,897,793 sequences after filtering. The mean number of sequences obtained per sample was 61,960 (maximum [max], 205,545; and minimum [min], 2,751). The mean number of sequences per sOTU was 5,420 (max, 2,389,487; min, 10). To determine the sequence depth for further analysis, alpha diversity rarefaction analysis was performed rarefied to 50,000 sequences (data not shown). The analysis showed that a sampling depth of approximately 10,000 was sufficient to maintain the diversity in our samples. Further analyses were therefore rarefied to 10,000 sequences resulting in the exclusion of three samples. These three samples were also filtered out from the final table, resulting in a table with 302 samples with a total of 18,880,504 sequences (mean, 54,391; min, 11,194). An overview of the final sample set is shown in Table 1.

**TABLE 1:** Number of samples per sample type included in the final data set^*a*^

Country	Sponge	Cloth	Sink	Cutting board	Tap handle	Counter top	Handles^*b*^	Total
France	14	1	14	9	10	13	5	66
Hungary	0	0	3	5	8	8	5	29
Norway	1	10	14	4	14	9	11	63
Portugal	10	11	7	5	13	12	6	64
Romania	15	10	13	12	8	11	11	80
Total	40	32	51	35	53	53	38	302

a There are 74 different households in the study (14 to 15 households per country) in total; of these, 67 had more than one sample passing the selection criteria.

b Handles are a pooled sample of hand contact points (kitchen cabinet handles, drawer handles, and refrigerator door handle).

### Overall bacterial composition in kitchens.

**FIG 1:**
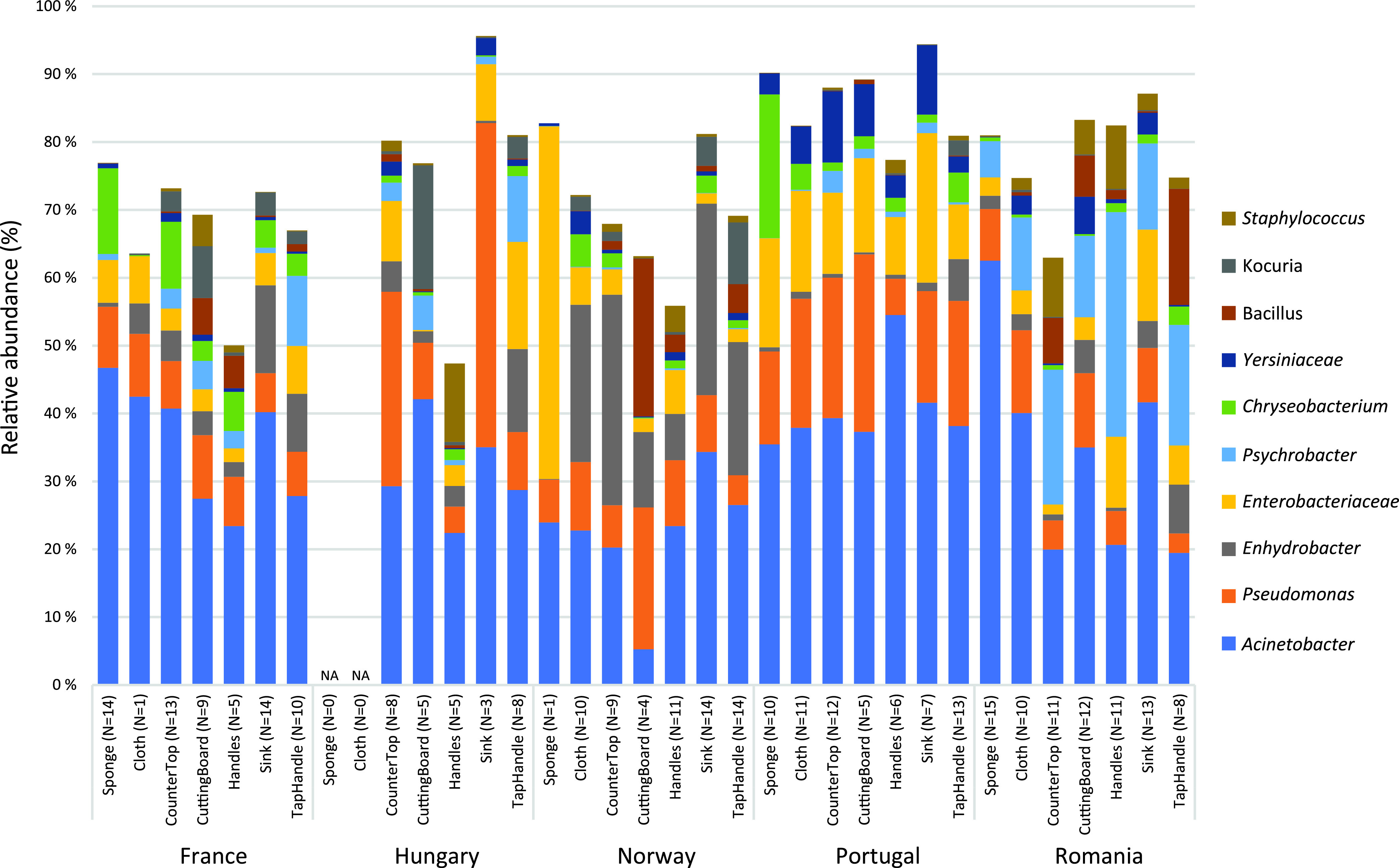
Relative abundance (%) of the 10 most abundant genera/families in the different sample types and countries. The values are based on the average of similar sample types within each country. The number of samples (N) representing each bar is written in parentheses after the sample type. There were no sponges or cloths from Hungary (marked NA in the figure).

According to the classification of Silva, the kitchen microbial community had members from 31 phyla, 62 classes, 155 orders, 297 families, 793 genera, and 3,487 sOTUs. The most frequently occurring sequences belonged to the phyla *Proteobacteria* (80%), *Firmicutes* (10%), *Bacteroidota* (5%), and *Actinobacteria* (4%). The most abundant genera/families (average relative abundance above 1% across all samples, L6 table [see Materials and Methods]) were Acinetobacter (34.2%), Pseudomonas (10.7%), *Enhydrobacter* (7.3%), *Enterobacteriaceae* (7.1%), *Psychrobacter* (5.5%), *Chryseobacterium* (3.5%), *Yersiniaceae* (2.3%), *Bacillus* (2.1%), *Kocuria* (1.9%), Staphylococcus (1.8%), *Pantoea* (1.5%), and Streptococcus (1.2%) (see File S1 in the supplemental material). Figure 1 shows the relative abundance of the most frequent genera/families in the different sample types and countries.

To get an overview of the number of sOTUs of the 10 most abundant genera/families, the complete sOTU table (before filtration) was investigated. There were 45 different sOTUs within the Acinetobacter genus; however, only 17 had a relative abundance above the filtration threshold (see Materials and Methods). The most frequent Acinetobacter species was *A. johnsonii* (mean relative abundance of 11.9%), followed by a species (10.2%) that could not be identified by sequence search (best match, KNN1) in Ribosomal Database Project (RDP) 11. Pseudomonas was represented by 64 sOTUs, where 20 had a relative abundance above the threshold. The sOTU with the highest mean relative abundance (3.6%) accounted for approximately one-third of the sOTUs in the Pseudomonas genus. Due to the highly diverse phylogeny of Pseudomonas (21), no further sequence search to identify to species level was attempted. There were three sOTUs assigned to *Enhydrobacter*, one of which was above the filtration threshold. The most abundant sOTU (7.2% mean relative abundance) had sequence similarity with both Moraxella osloensis and Enhydrobacter aerosaccus (BLASTnt and RDP). There were 19 sOTUs within the *Enterobacteriaceae* family, 8 of which were above the filtration threshold. Among these, one sOTU had the closest sequence match with Salmonella enterica (RDP 11 search and BLASTnt search). *Psychrobacter*, *Chryseobacterium*, *Yersiniaceae*, *Bacillus*, *Kocuria*, and Staphylococcus were represented by 21, 67, 3, 41, 16, and 12 sOTUs, respectively. For more details see File S2.

**(i) Potential pathogens.** There is a concern that the kitchen can act as a reservoir for pathogenic bacteria originating from raw food products prepared in the kitchen. Cultivation using enrichment in selective media in a previous study on the same samples suggested that the presence of the food-associated pathogens Campylobacter and Salmonella was relatively rare in kitchens (22). Only 40 to 75% (depending on the pathogen) of samples in the present study were tested for Campylobacter, Salmonella, and *Listeria* using standard methodology with preenrichment and selective media. Of these, none were positive for Salmonella, one was positive for Campylobacter (cloth), and 16 samples were positive for *Listeria* (sponges, sinks, and cloths). Although the sequencing technique used in this study lacks the phylogenetic resolution to detect pathogens, and relatedness based on partial 16S rRNA gene sequences is only a weak indicator of the pathogenic potential of the identified bacteria, it is of interest to look at the families/genera that could include potential pathogens or indicators of fecal contamination. Table 2 shows the relative abundance of four sOTUs assigned to *Enterobacteriaceae* (sequence similarity to S. enterica), Campylobacter jejuni, Escherichia/*Shigella*, and *Listeria*. The table shows that the sOTU related to S. enterica (*Enterobacteriaceae*) was present in all countries—16.2% of all households and 4.6% of all samples. This sOTU had a low relative abundance in all samples (mean value of 0.2%) except for one Norwegian sponge sample, where it had a relative abundance of 43%. Escherichia/*Shigella* was also present in samples from all countries. Campylobacter jejuni was only present in samples from France, Portugal, and Romania. Most of these potential pathogens had relatively low abundance. For more details see File S2. In addition to the pathogens discussed above, opportunistic pathogens could also present a concern. Species within the genera Acinetobacter, *Chryseobacterium*, and *Moraxella*/*Enhydrobacter* have been referred to as belonging to risk group 2 species (moderate individual risk, low community risk) (1, 14).

**TABLE 2:** Relative abundances and occurrences of taxa (sOTUs) associated with potential pathogens and fecal indicators^*a*^

Taxa (sOTU)	Relative abundance (%)		Occurrence (%)		Occurrence in households/samples per country				
	Mean	Max	Samples (302)	Households (74)	France (15/66)	Hungary (14/29)	Norway (15/63)	Portugal (15/64)	Romania (15/80)
*Salmonella enterica* ^*b*^	0.23	43	4.6	16	2/2	2/2	4/6	1/1	3/3
*Escherichia-Shigella*	0.038	7	14	37	9/13	3/3	5/5	2/3	8/17
*Campylobacter jejuni*	0.0018	0.24	6	22	7/9	0	0	3/3	6/6
*Listeria*	0.00023	0.062	0.66	2.7	0	2/2	0	0	0

a The data was collected from the unfiltered sOTU table. The occurrence of each taxon is given in percentage of the total number of samples (302) and households (74), and as the number of occurrences per household and samples (number of households/number of samples) per country. The numbers in parentheses for each country are the total number of households and samples in that country.

b sOTU assigned to *Enterobacteriaceae* according to SILVA classification and related to *Salmonella enterica* according to a search in RDP11 and BLASTnt (assessed June 21st 2022).

### Differences in bacterial community structure.

**(i) Bacterial diversity within sample groups (alpha diversity).** A total of 3,487 sOTUs were detected in the kitchen samples. Different metrics can be used to estimate the alpha diversity (the mean species diversity in a site at a local scale), e.g., observed features (sOTUs), Shannon index, and Faith’s phylogenetic diversity (faith_pd). The number of observed sOTUs varied within the different samples (min, 19; max, 468; average, 133). The alpha diversity was significantly different (*P* < 0.0001) between countries and between sample types for all tested metrics.

**FIG 2:**
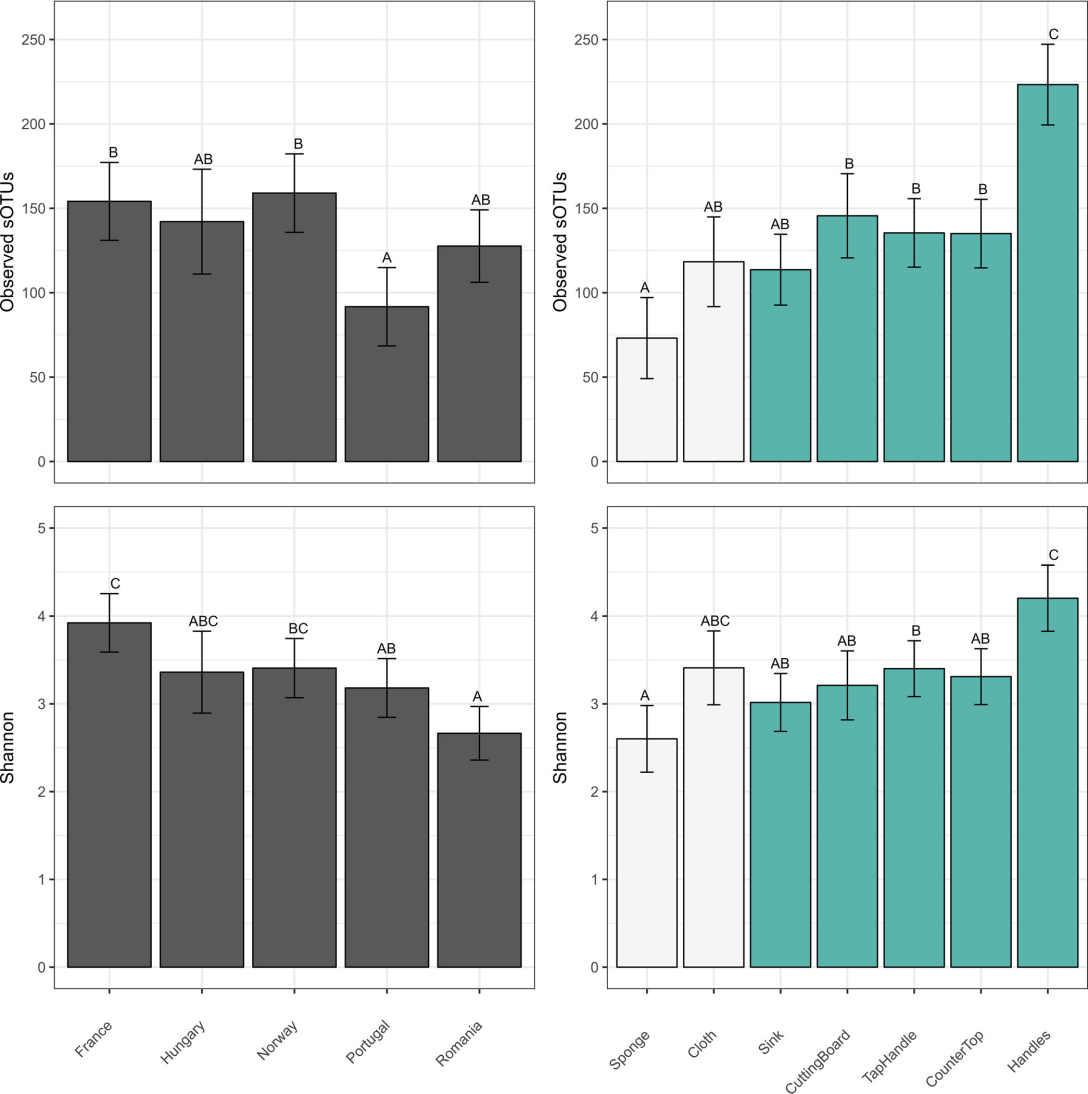
Bacterial diversity (alpha diversity) measured by observed features (observed sOTUs) (top two panels) and Shannon diversity (bottom two panels). Alpha diversity is measured per country (left) and per sample type (right). The alpha diversity is presented as the estimated marginal means for observed features (observed sOTUs) and Shannon diversity. Different colors are used to contrast cleaning utensils (sponge and cloth) and surface samples. The error bars show the 95% confidence intervals. Different letters indicate statistically significant differences (*P* < 0.05).

Figure 2 shows that Portugal had significantly fewer observed sOTUs (average of 86) than France (average of 145) and Norway (average of 165) (*P* < 0.05), while France, Hungary, and Norway had similar diversity levels. Similar results were obtained with faith-pd (results not shown). When applying the Shannon index (richness and evenness), however, the alpha diversity decreased in the following order: France, Norway, Hungary, Portugal, Romania. Romania was significantly lower than France and Norway (Fig. 2). If restricting the analysis to cleaning utensils only, country differences are less evident (*P* < 0.05 for observed sOTUs and faith_pd but not Shannon index), whereas when restricting the analysis to surfaces, the country differences remain significant (*P* < 0.01 for all three metrics).

The largest alpha diversity (observed sOTUs) was observed for handles (average of 226), which was significantly higher than that of all other sample types, and the lowest was observed for sponges (average of 67), which was significantly or near significantly lower than that for all other sample types (Fig. 2). There were no significant differences in alpha diversity between the other sample types. Similar results were obtained for the three metrics (faith_pd results not shown).

**FIG 3:**
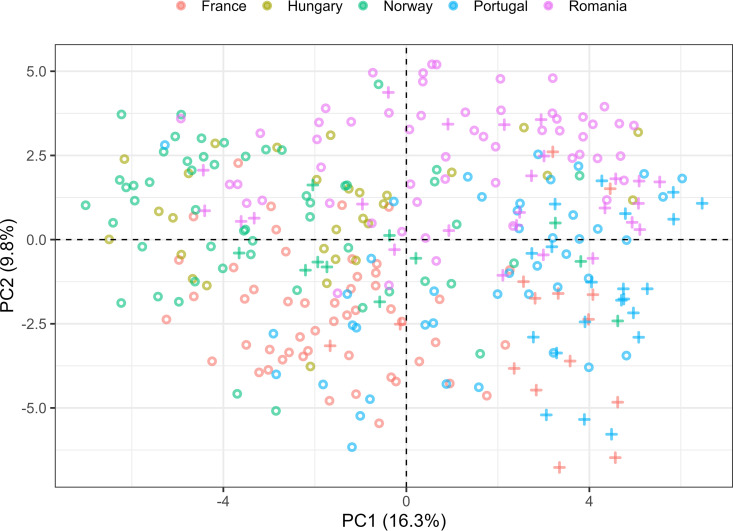
Principal-component analysis (PCA) of L6 (genus level) data. The analysis was performed on CLR transformed data at the L6 level after filtering. The samples are colored based on country and symbols representing either cleaning utensils (crosses) or surface samples (circles).

**(ii) Between-sample variation.** Several comparisons were made initially, e.g., comparing consumer groups, income, etc., and the most interesting findings are presented. Principal-component analysis (PCA) of the filtered level 6 (genus) table shows a clear tendency of samples grouping according to country and sample category (cleaning utensils and surface sample) (Fig. 3). Within each country there were several households, and we observed that some of the samples from the same household were close together, whereas others were more distant. However, household differences were also related to household size and composition (families, young adults, elderly), and no effects of these factors were found in the initial analyses.

Beta diversity analyses (based on all sOTUs) were also performed comparing three different metrics (Jaccard, Bray-Curtis, and UniFrac [weighted and unweighted]), where weighted UniFrac analysis resulted in the principal-coordinate analysis (PCoA) plot with the highest explained variance (axis 1, 22.9%; axis 2, 15.76%). Since weighted UniFrac incorporates and emphasizes the phylogenetic relationships among features as well as abundance, this metric was used to illustrate the clustering of the microbiota of all samples visualized by countries, sample categories (cleaning utensils and surface samples), and sample types (Fig. S1). The PCoA plots indicate that there were both country- and sample-specific differences in the microbiota, although the separation between countries or sample categories was not as clear as that for the filtered L6 data (Fig. 3).

### In-depth analysis of taxonomic similarities and differences.

To further investigate the bacterial similarities and difference between countries and sample types, more detailed analyses were performed on central log transform (CLR) data at L6 after filtering (as described in Materials and Methods) to focus on the most abundant genera.

**FIG 4:**
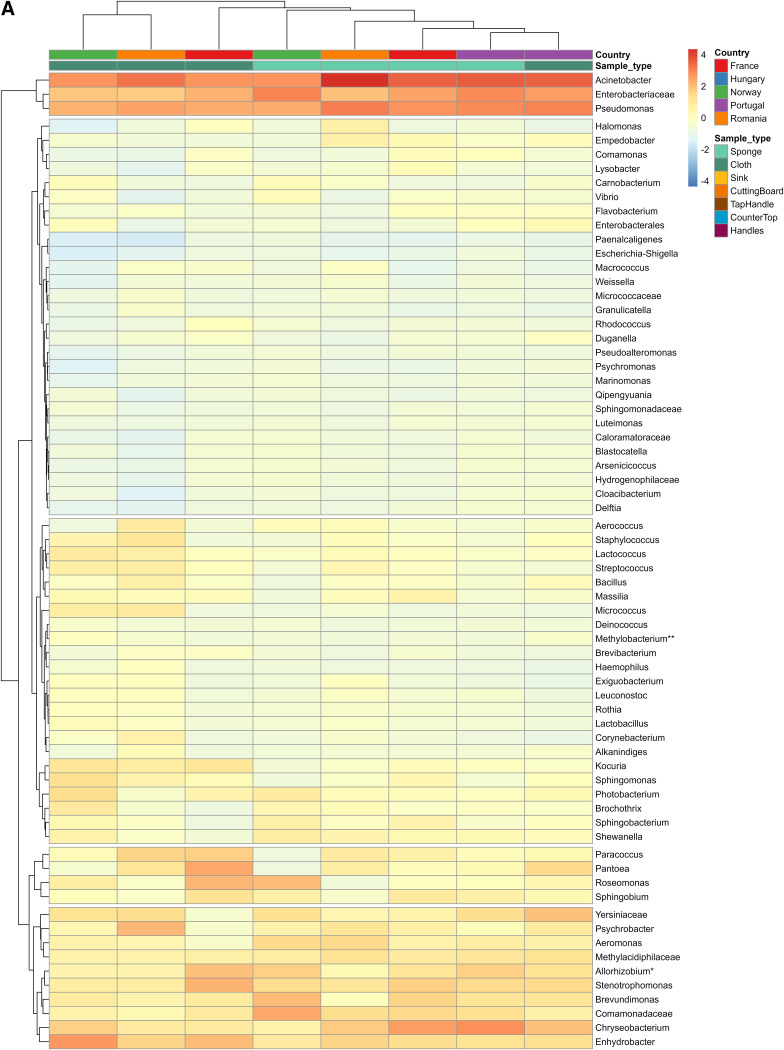

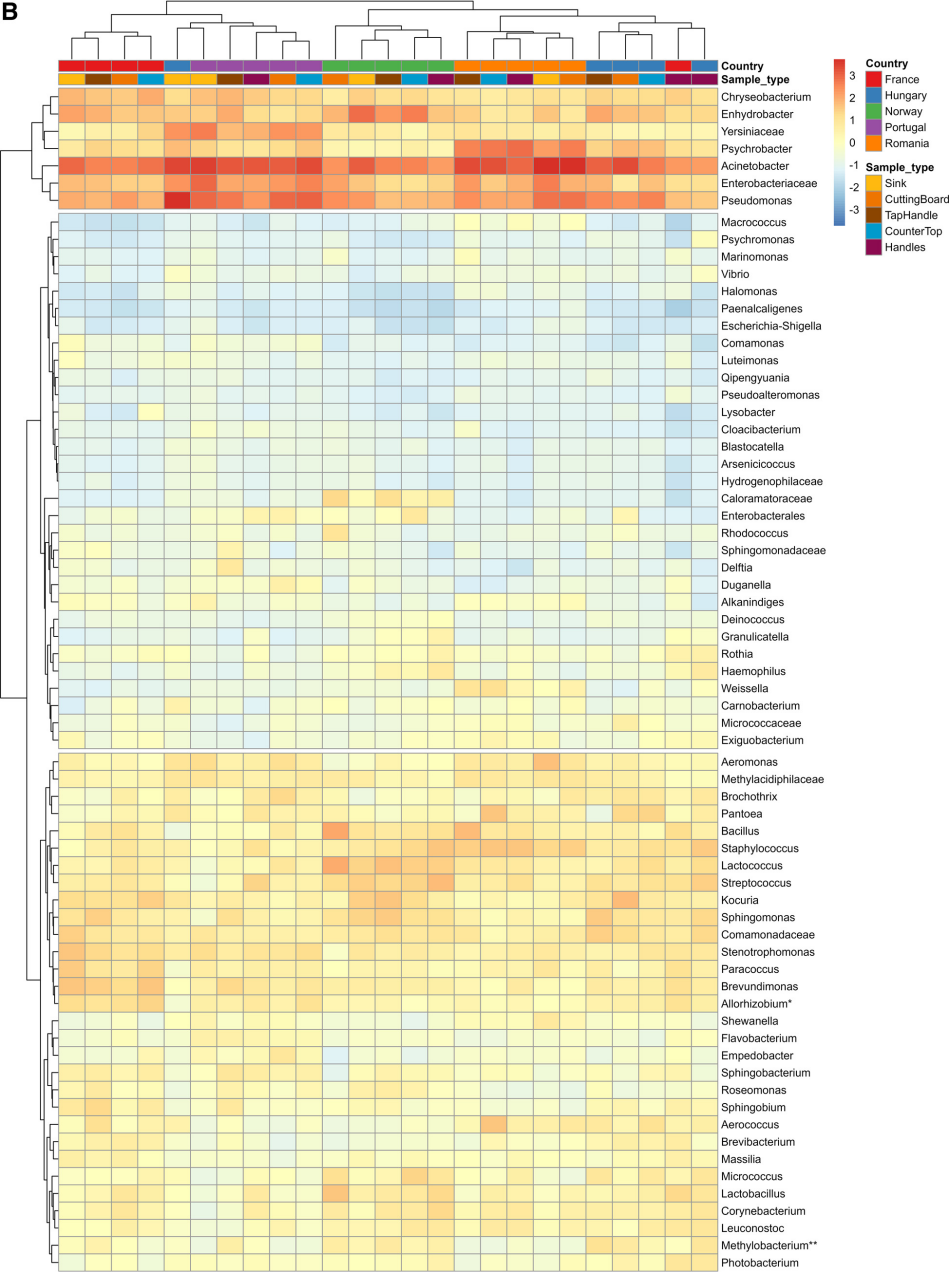
(A and B) Heatmaps of the bacterial genera with average relative abundance higher than 1% or exceeding 5% in at least one sample (*N* 68 genera) for cleaning utensils (A) and surface samples (B). The heatmaps are based on the average of a sample type within a country. Allorhizobium*, *Allorhizobium*-*Neorhizobium*-*Pararhizobium*-*Rhizobium*; Methylobacterium**, *Methylobacterium*-*Methylorubrum*.

The PCA plot and the heatmap of all samples (Fig. S2) show a tendency for the microbiota to cluster according to sample category (surface or cleaning utensils) as well as country. Separate heatmaps were therefore generated for surface and cleaning utensil samples. To better illustrate the clustering of bacterial taxa between country and sample types, heatmaps were produced from averages of each sample type within the countries (Fig. 4A and B).

Figure 4A shows that although the microbiota in sponges and cloths were relatively similar, the microbiota were more similar across countries than between sponges and cloths, except for sponges and cloths from Portugal that clustered together. The most common genera (Acinetobacter, *Enterobacteriaceae*, and Pseudomonas) clustered together based on the similarity of their relative abundance pattern across all samples.

Figure 4B shows that the surface samples with few exceptions clustered according to country, meaning that the microbiota of the different surface samples were more similar within one country than specific surface samples across countries. There were some exceptions from this country-of-origin clustering: sink samples from Hungary were more similar to sink samples from Portugal than to other surface samples from Hungary; handles from France were more similar to handles from Hungary than to the other surface samples from France. The figure also shows that the microbiota of surface samples in France were more similar to surface samples in Portugal than to the other countries and that the microbiota of surface samples from Romania were most similar to samples from Hungary. Similar to cleaning utensils, most highly abundant genera clustered together based on the similarity of their relative abundance pattern across all samples: *Chryseobacterium*, *Enhydrobacter*, *Yersiniaceae*, *Psychrobacter*, Acinetobacter, *Enterobacteriaceae*, and Pseudomonas.

**FIG 5:**
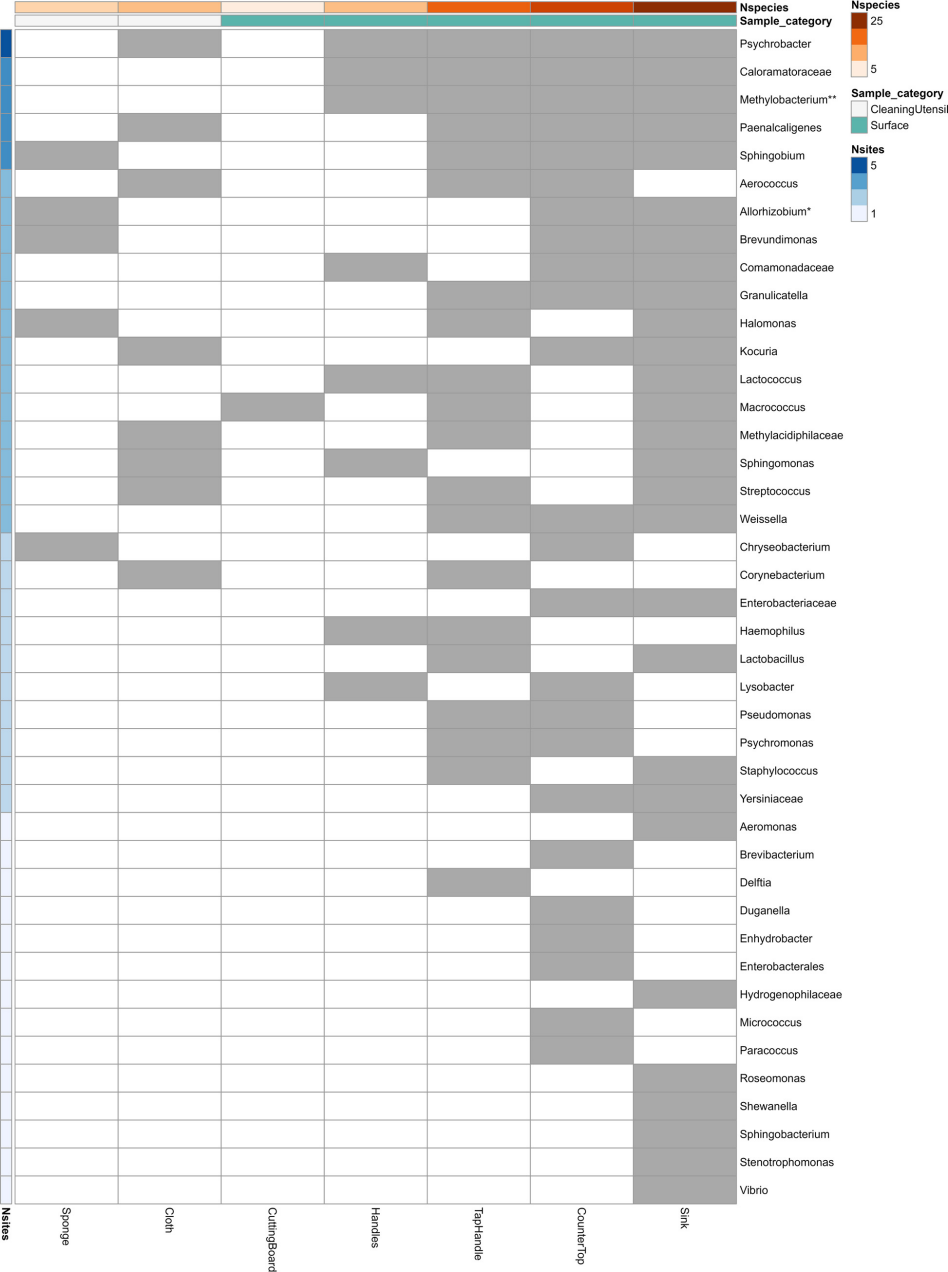
Bacteria with significantly different relative abundance between countries for various sample types. Gray boxes indicate sample types/species for which there are significant (FDR-adjusted *P* value of <0.01) differences between countries. The number of sample types with significant differences between countries is indicated on the left side (Nsites), whereas the number of genera significant for each sample point (Nspecies) is indicated on the top together with the sample category.

**(i) Differences between countries.** In addition to identifying bacteria with similar relative abundance patterns across countries and sample types, we also wanted to identify if there were bacteria with different relative abundances across countries. A total of 42 genera/families had significantly different relative abundance between countries for various sample types (Fig. 5). As expected from the results of the heatmap analyses (Fig. 4), only a few bacteria were significantly different between countries for sponges and cloths compared to surface samples (except for cutting board). The explained variance for the country effect was also lowest for sponges (13%) and highest for handles (25%) (Table S1). Sink samples had the highest number of bacteria with significantly different relative abundance between countries, followed by countertop and tap handle samples. The genus that differed significantly in relative abundance between countries for most sample types was *Psychrobacter*.

### Core microbiota: occurrence and abundance.

**FIG 6:**
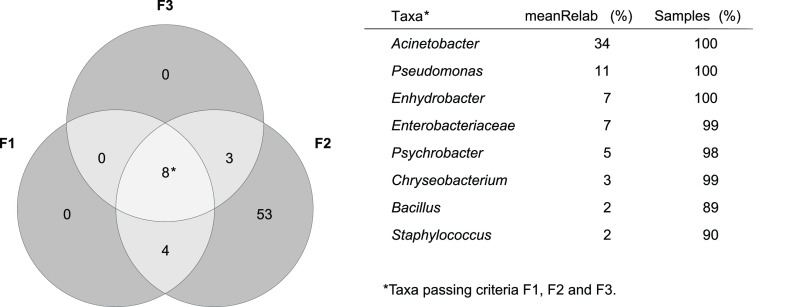
Venn diagram of bacterial taxa and core microbiota of kitchens. The diagram is based on the L6 table and different criteria: F1, arithmetic mean abundance above a threshold of 1%; F2, MeanMax (arithmetic mean abundance above a threshold of 1% or an abundance above 5% in at least one sample); F3, occurrence (the taxa is present in at least one sample for all consumers). The core taxa are listed together with the meanRelab (mean relative abundance [%]) and samples (proportion [%] of samples where the taxa are present). The four taxa fulfilling criteria F1 and F2 (not F3) were *Yersiniaceae*, *Kocuria*, *Pantoea*, and *Streptococcus*, while the three taxa fulfilling criteria F2 and F3 (not F1) were *Aeromonas*, *Comamonadaceae*, and *Allorhizobium*-*Neorhizobium*-*Pararhizobium*-*Rhizobium*.

Although differences in the microbiota were related to sample category, country of origin, and sample type, a small group of bacteria was present in most samples. To determine if a bacterial taxon was part of the core microbiota, different filtration criteria were explored. Filtering based on relative abundance gave a different set of cores than filtering based on occurrence. When looking at the number of taxa passing different thresholds for mean relative abundance, we observed that the number of taxa passing the filter decreased rapidly between 0.01% and 1%, which indicates that if we want to consider the most abundant genera, a natural threshold is around 1%. Figure 6 is a Venn diagram based on the L6 table (genera) comparing three different filtrations: F1, mean (arithmetic mean abundance above a threshold of 1%); F2, MeanMax (arithmetic mean abundance above a threshold of 1% or abundance above 5% in at least one sample); F3, occurrence (taxa present in all consumers in at least one sample). Filtration based on mean and maximum abundance (F2) resulted in the highest number of bacteria (68 different taxa), while filtering based on a mean threshold (F1) or occurrence only (F3) resulted in 12 and 11 taxa, respectively. That is, 53 bacterial taxa were only highly abundant in one or a few samples. Only eight taxa passed all three criteria and were interpreted as the core microbiota (Fig. 6). Of these, the three bacterial taxa Acinetobacter, Pseudomonas, and *Enhydrobacter* were present in all samples and were also the taxa with the highest mean relative abundance. For an overview of the other taxa in the Venn diagram, see Table S2. Similar core analysis was performed on the sOTU table, resulting in 3 core sOTUs: *Enhydrobacter*, *Enterobacteriaceae*, and Pseudomonas (see Fig. S3 and Table S3). Only one sOTU, assigned to *Enhydrobacter*, was present in all samples.

**FIG 7:**
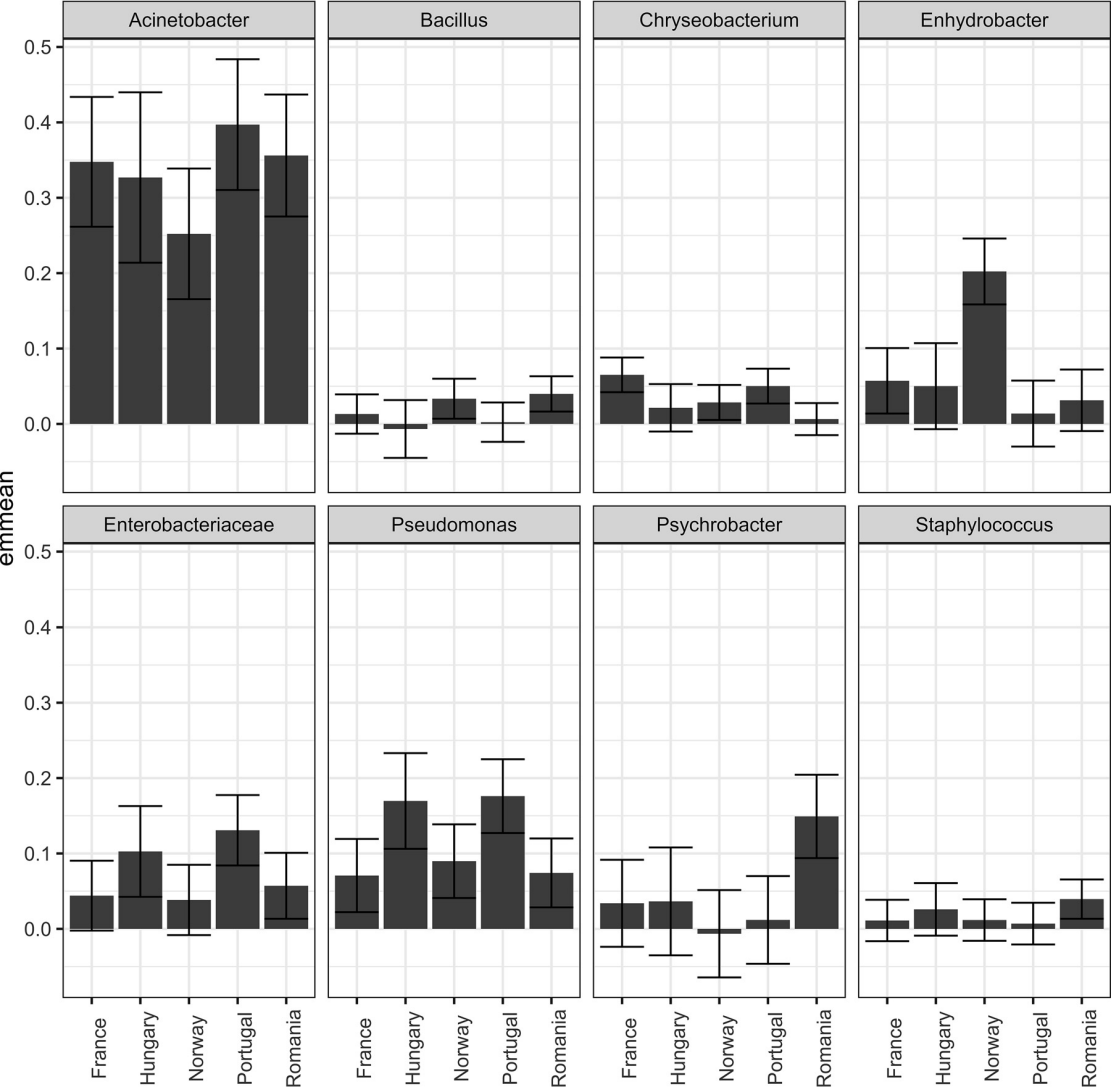
Estimated marginal means of the relative abundance for each of the taxa defined as core microbiota. The error bars represent the confidence interval (95%).

**FIG 8:**
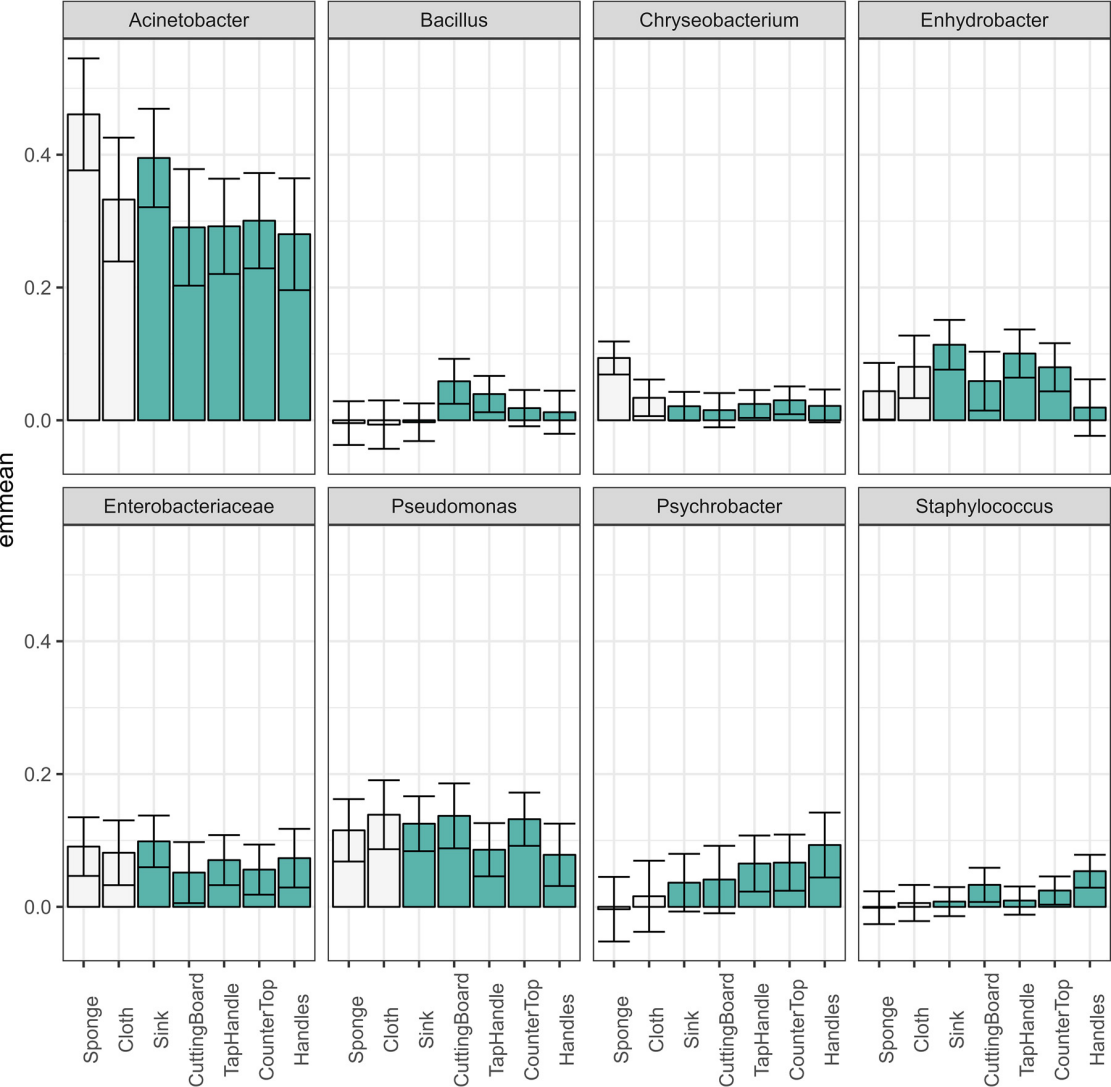
Estimated marginal means of the relative abundance for each of the taxa defined as core microbiota. The error bars represent the confidence interval (95%). White bars represent cleaning utensils and green bars for surface samples.

The eight bacterial taxa identified as belonging to the core microbiota varied in relative abundance between countries and sample types. Five of these (Pseudomonas, *Enterobacteriaceae*, *Enhydrobacter*, *Psychrobacter*, and *Chryseobacterium*) had significantly different relative abundances between countries (Fig. 5). The variance component estimated for the household effect varied considerably for the eight taxa. This was very low for *Bacillus* (1.3%), low for *Chryseobacterium* (11.0%), and high for *Psychrobacter* (31.5%), showing that variability between households depends strongly on the taxa investigated. Figures 7 and 8 show the relative abundance of the eight core bacteria between different countries and sample types, respectively.

Acinetobacter was significantly different between sample types (*P* < 0.01), and although the abundance was relatively high in all samples, highest abundances were observed in humid samples such sponges, cloths, and sinks. Pseudomonas was significantly different between countries (*P* < 0.005), with the highest abundances in samples from Portugal and Hungary. *Enterobacteriaceae* was significantly different between countries (*P* < 0.05) and had the highest abundances in samples from Portugal and Hungary. *Enhydrobacter* was significantly different between countries (*P* < 0.0000005) and sample types (*P* < 0.01) and had relatively low abundance in all countries except for in Norwegian samples. *Enhydrobacter* had a higher relative abundance in tap water-related samples such as sink and tap handles. *Psychrobacter* was significantly different between countries (*P* < 0.005) and sample types (*P* < 0.05), with the highest relative abundance in samples from Romania and the lowest abundance in cleaning utensil samples (sponges and cloths). *Chryseobacterium* had significantly different relative abundances between countries (*P* < 0.005) and sample types (*P* < 0.0001), with the highest relative abundances in samples from France and Portugal and in sponges. *Bacillus* was significantly different between sample types (*P* < 0.05), with the lowest relative abundances in humid samples such as sponges, cloths, and sinks. Staphylococcus was significantly different between sample types (*P* < 0.05) and had the highest relative abundance on handles.

## DISCUSSION

To the best of our knowledge, this is the first study that has characterized the bacterial community of kitchen surfaces and cleaning utensils across several households and countries and identified a core microbiota. Although the households in the current study had considerable differences with respect to kitchen standards (households with and without running water, indoor sink, and dish washing machine), food preparation routines, and dietary habits (23–25), we still found a set of core bacteria resident in the kitchen environment. Also, similar bacteria were found in European kitchens as were previously found in other kitchen surfaces and kitchen sponge studies (2, 7, 12–15).

Based on abundance and occurrence, we identified eight genera/families, Acinetobacter, Pseudomonas, *Enhydrobacter*, *Enterobacteriaceae*, *Psychrobacter*, *Chryseobacterium*, *Bacillus*, and Staphylococcus, that represent the core microbiota in European kitchens. At the sOTU level, only three core bacteria were identified (assigned to *Enhydrobacter*, *Enterobacteriaceae*, and Pseudomonas). The number of core bacteria is difficult to compare with other studies due to the lack of consensus in scientific papers about how a core microbiome should be defined in practice. Different definitions are used in the literature to define a core microbiota: occurrence and/or abundance of a bacterium (including or excluding genomic or functional attributes). Neu et al. (26) listed three main methods used in the assessed studies for calculating the core microbiome: occurrence only, relative abundance only, or a combination of abundance and occurrence. They found that in most studies the core microbiomes were quantified based solely on the occurrence of a taxon (OTU, genus, etc.), for instance with a threshold on the proportion of the samples collected. That said, a similar number of core bacteria (based on abundance and occurrence) were identified in four health care-associated institutes in Taiwan (27), where nine core genera (seven associated with human skin) were identified. Three of these genera were in common with the core kitchen bacteria (Acinetobacter, Pseudomonas, and Staphylococcus) identified in the present study and were, together with Enterobacter, regarded as health care-associated pathogens. The number of core bacteria was also comparable to a study conducted in a cooking center for hospital foodservice (food preprocessing rooms, storage room, and kitchen) (28), where 14 core OTUs (based on an occurrence of >70%) were identified, with *A. johnsonii* being the most abundant OTU. Of the 14 core OTUs, Acinetobacter and *Chryseobacterium* were in common with the core bacteria identified in the present study. A higher number of OTUs, 32 were reported to be shared by the kitchen, storage area, and tools, with *A. johnsonii*, *Alicyclobacillus*, Acinetobacter spp., and *Chryseobacterium* being the most abundant (28). The reason for the low number of common core bacteria may be related to the large difference in cleaning and disinfection routines between a hospital cooking center and household kitchens, as well as differences in geographical range, where the present study covers a larger geographical range. Other reasons may be differences in methodology (PCR primers, taxonomic databases, etc.) as well as different definitions of the core microbiota.

Common for the present and the above-mentioned studies is that Acinetobacter was part of the core microbiota. We found that Acinetobacter was present in all countries and samples and that its relative abundance was high (especially in humid samples such as sponges, cloths, and sinks) and not significantly different between countries. We have previously also reported that Acinetobacter was among the most dominant bacteria in used sponges and brushes across different countries in Europe (12), and Adams et al. (2) reported that Acinetobacter was abundant in kitchens regardless of geographic location (South Korea, Colorado, North Carolina). One explanation for the dominance of Acinetobacter in kitchens could be that it probably enters the kitchen daily, since it is highly occurrent on vegetables, meat, fish, and milk and appears to be persistent in drinking water systems (29–31). Acinetobacter spp. are also commonly found in food processing environments (32), especially in fish processing environments (33), which are often humid. Although (Acinetobacter johnsonii, A. pittii, and *A. ursingii*), together with Chryseobacterium hominis and Moraxella osloensis, have been referred to as belonging to risk group 2 species (moderate individual risk, low community risk) (1, 14), these bacteria are generally not regarded as a threat to the public.

In addition to Acinetobacter, *Enhydrobacter* and Pseudomonas have previously also been reported to be closely associated with kitchens (2), as well as *Chryseobacterium* in kitchen sponges (13). Cardinale et al. (14) showed that kitchen sponges revealed massive colonization by Acinetobacter, *Moraxella* (Moraxella osloensis), and *Chryseobacterium* species, and we have previously reported a common set of bacteria in Norwegian and Portuguese brushes and sponges, dominated by Acinetobacter, *Chryseobacterium*, *Enhydrobacter*, *Enterobacteriaceae*, and Pseudomonas (12). This close correlation with sponge microbiota and overall kitchen microbiota may in part be explained by the high bacterial load in sponges and the hypothesis that kitchen sponges massively absorb and spread microorganisms, leading to contamination of kitchen appliances, surfaces, and food (13). *Chryseobacterium* has been found, in addition to kitchen surfaces and cleaning utensils, in salmon and small-scale cheese production environments and in raw foods (34–37).

*Enhydrobacter* has previously been reported among the dominant bacteria in Norwegian kitchen sinks (19) and among the core microbiota in sponges/brushes (12). The representative sequence of the sOTU assigned to *Enhydrobacter* in the current and above-mentioned studies had sequence similarity to both Moraxella osloensis and Enhydrobacter aerosaccus. Moraxella osloensis is a human commensal organism that rarely causes infections other than in immunocompromised individuals (38). In both this and in our previous study, *Enhydrobacter* had a higher abundance in Norwegian samples than in samples from the other European countries; however, in Møretrø et al. (12) the lower relative abundance in Portuguese items compared to Norwegian items was mainly due to a lower abundance in sponges than in brushes. In Norway the most common cleaning utensil for washing up is a brush (11). Brushes are shown to dry more quickly than sponges and have a lower bacterial load than sponges (12). In the current study, the highest abundance of *Enhydrobacter* was in sinks and tap handles.

Both Staphylococcus and *Bacillus* had low relative abundance in moist samples. The highest relative abundance of Staphylococcus was found on handles. This may be explained by Staphylococcus being frequently transferred from hands upon contact with handles, as Staphylococcus spp. are a major part of the resident flora of human skin (39). The observed lower relative abundance of *Bacillus* in humid sample types than in sample types that are usually drier, may be explained by *Bacillus* likely being outcompeted by other, fast-growing bacteria in humid niches but, through its formation of endospores, surviving better than other bacteria in dry environments.

*Enterobacteriaceae* consists of many genera and species, some of which can have pathogenic potential. However, the pathogenic potential of a bacteria is often species dependent and sometimes strain dependent, and the method used in this study does not have the resolution needed to identify the bacteria to the species level and/or to evaluate, for example, the presence/absence of virulence factors. Reviewing the residential bacteria on surfaces in the food industry, Møretrø and Langsrud (33) reported that *Psychrobacter* spp. were among 12 taxa composing >10% of the bacteria identified in at least one processing plant. *Psychrobacter* species are primarily isolated from seawater, fish, and chilled meat (40), and *Psychrobacter* together with Acinetobacter and Pseudomonas were also found to be abundant in several samples from a hospital foodservice cooking center and were suggested to be members of the resident microbiota of processing plants (28).

Of the eight core kitchen bacteria identified, all but *Enhydrobacter* have been shown to be common in the food industry (33, 41, 42). Pseudomonas and Acinetobacter are among the most isolated bacteria in relatively humid food processing environments and are also frequently isolated from foods (33). Many are also widely distributed in nature (e.g., soil and water), have low nutrient requirements, grow fast at a wide temperature range, and may form biofilms (33). Members of the Pseudomonas genus (P. fluorescens group, along with the psychrotrophic Pseudomonas fragi, P. lundensis, and P. putida) can be involved in food spoilage and are often isolated from aerobically spoiled meat even during storage at low temperatures (43–45).

In addition to identifying a core microbiota, we also observed a tendency of similarities within countries for kitchen surfaces. Others have found that the kitchen surface communities, in a small geographic area, in general differed based on the kitchen of origin, indicating that communities from the same kitchens were more similar than communities from different kitchens (7). A meta-analysis published by Adams et al., (2) indicated a site-level similarity across residential surfaces for bacteria. Until now, studies have been lacking a large collection of households and sample sites across a wide geographic space to explore the variation of the kitchen microbiota by household. We found similarities between countries from the same regions in Europe. We observed that surface samples from neighboring countries, Romania and Hungary, grouped together based on the microbial pattern. Similarly, surface samples from the southern part of Europe, France and Portugal, grouped together. These similarities could be related to climate, household demographics, and/or dietary differences, but further investigations need to be performed to determine this. In contrast to surface samples, the microbial pattern of cleaning utensils seemed to be more influenced by the type of cleaning utensil than country of origin, possibly reflected by the low bacterial diversity.

We found that the alpha diversity (observed sOTUs) was comparable to previous results on cleaning utensils (13, 14) and kitchen surfaces (7, 15) but low compared to, e.g., soil, sediment, and water, as expected (46). The microbiota was least diverse in sponges, as also found by Flores et al. (7) (only one sponge), while it was most diverse on handles, as also found by Hodgetts et al. (47). Handles are dry hand contact points, and one can speculate that the higher diversity is caused by the variation of bacteria transferred from hands of the household’s occupants as well as a higher presence of dead bacteria compared to moist surfaces, where a selection of bacteria will grow and dominate. In general, sponges have a high bacterial load, where a few types of bacteria grow extremely well and outcompete the others.

There is a concern that sponges can be the cause of cross-contamination of pathogens, and we have previously shown that, e.g., Salmonella can survive for more than 7 days in some sponges (11). DNA from a wide range of foodborne pathogens, including Salmonella enterica has previously been detected in sponges from low-income households (15). The sequencing technique used in this study does not have the phylogenetic resolution to detect pathogens, but we identified sOTUs that may be associated with pathogens. These sOTUs were low in abundance but broadly distributed throughout the households, corresponding to previous studies by Flores et al. (7). Of special interest was one sOTU with sequence similarity to Salmonella enterica that was present in all countries and 5% of the samples. Although at a relative low abundance in most samples, this sOTU dominated one Norwegian sponge, illustrating the potential risk of cross-contamination of pathogens from sponges if present. This particular sponge, as well as some of the other samples in the current study, have previously been analyzed by a culturing-dependent method with respect to Salmonella, and none of the cleaning utensils or surface samples were positive for Salmonella (22). The sequences identified may therefore originate from dead bacteria.

This study shows that there is a small number of bacterial taxa that compose a core kitchen microbiota despite considerable variations in kitchen standards, kitchen practices, cleaning regimes, households, and dietary differences. In addition to finding a core microbiota across countries and sample types, we also show that the microbiota on kitchen surface samples, on average, were more similar within one country than within a surface sample type across countries. For cleaning utensils, we observed that the microbiota was more closely related to type of cleaning utensil, sponge or cloth, than country of origin.

In conclusion, this study has contributed to deepening our understanding of the kitchen microbial community. Follow-up studies are needed to correlate these findings with food safety behaviors and the potential impact on human health.

## MATERIALS AND METHODS

### Consumer visits and sampling.

This study was performed as part of a larger study in which domestic kitchens in Europe were visited and microbial analyses and observational studies were performed. Details about the visits, households, recruitment procedures, and the findings of pathogens have been described previously (22). Briefly, the households covered three consumer groups assumed to cover different risk groups based on behavior and vulnerability: young single men, pregnant women/families with child <1 year old (<5 years for Portugal), and elderly people (>70 years). Households had different income levels and different levels of education and were in both rural and urban areas. The present study covers the microbiota from kitchen surfaces and cloths and sponges from 74 households in five European countries: France, Hungary, Norway, Portugal, and Romania.

Surface samples from different objects were taken from each kitchen: a cutting board, the countertop, the sink, a tap handle, and a pooled sample of hand contact points (kitchen cabinet handles, drawer handles, refrigerator door handle). Surface samples were taken before dinner preparation to reflect the kitchen resident microbiota and exclude fresh contamination from food. Cleaning utensils were collected after food preparation: a sponge, a cloth, or both, depending on what was used for cleaning kitchen surfaces in that household. Furthermore, only surface samples with total viable counts (TVC) over 2 log CFU/cm^2^ (based on limited sensitivity of the qualitative identification method used) were included. Approximately 20% of the surface samples had a TVC below this criterion. Additionally, approximately 20% of the samples with a TVC above the criterium failed to generate enough DNA for sequencing after PCR. Due to these criteria, the number of analyzed samples per sample type and country varied.

Surfaces were sampled by swabbing, using a prewetted cloth (product no. 4030/4031), SodiBox (www.sodibox.com) as described previously (22). All samples were placed in a cooling bag and transported to the laboratory. Within 1 to 5 h, the samples were placed at 4 to 6°C for 12 to 24 h before further analysis.

The sampling cloths and kitchen cloths/sponges were added to 25 mL buffered peptone water (BPW; bacteriological peptone; Oxoid Limited, Hampshire, UK) and other ingredients (Merck, Darmstadt, Germany) and subjected to homogenization (AES Laboratoire, Chemunex, Combourg, France) for 1 min. The enumeration of the total viable count (TVC) of the macerate was performed according to ISO 4833-1 (48). The bacterial counts were used only to select samples for DNA extraction as stated above.

A volume of 2 to 5 mL of homogenized suspension (as described above) was centrifuged at 13,000 × *g* for 5 min. Supernatants were removed, and pellets were stored at −20°C until analysis. Pellets from countries other than Norway were shipped to Nofima (Ås, Norway) for analysis. DNA was extracted from the pellets using the Qiagen DNeasy PowerSoil HTP96 kit including bead beating using FastPrep-96 (2 × 1,600 rpm, 1 min) and following the manufacturer’s protocol.

### Microbiota: 16S rRNA gene sequencing.

16S rRNA gene PCR (V4 region) and paired-end sequencing (2 × 150 bp) using the MiSeq reagent kit v3 on a MiSeq instrument (Illumina) were performed using the protocol presented by Caporaso et al. (49) as previously described (11).

The samples were sequenced together with other samples not included in this work and divided into eight different MiSeq runs. The sequences were processed in QIIME 2 (qiime2-2020.11) (50). Briefly, the data were demultiplexed using demux, paired ends were joined using vsearch, quality filtered based on a q-score above 30, and denoised using Deblur denoise-16S action, and taxonomy was achieved with classify-sklearn using the SILVA 16S database (Silva 138 99% OTUs from the 515F/806R region of sequences) (51–54). Deblur detected closely related bacterial sequences (sub-operational taxonomic units (sOTUs) with single nucleotide differences while removing false positives and maintaining stability in detection). After denoising with deblur 16S-action, 99.999% of the sequences were assigned to the domain Bacteria. The tables of representative sequences from each of the eight runs were then merged. Further, the merged table was filtered to remove any sequences of mitochondrial and/or chloroplast origin, resulting in the loss of 0 to 69% of the sequences (only two samples lost more than 50% of the sequences). On average, the samples retained 96.6% of the sequences. Cutting boards had the highest percentage of sequences of mitochondrial and/or chloroplast origin. Finally, the table was filtered to remove any sOTUs below 10 and sOTUs that were present in only a single sample. The taxonomy tables were collapsed to different levels: the level 6 (L6; genera) and sOTU table (feature table) were converted to relative values and exported to text files and further processed in Excel, Minitab, and R (55). Due to the low taxonomic resolution in some genera/families after SILVA classification, some representative sequences were subjected to further sequence match searching. Sequence match searching was performed using either Ribosomal Database Project (RDP) v11 (https://www.lcsciences.com/documents/sample_data/16S_sequencing/src/html/top1.html) or the BLAST blastn suite (https://blast.ncbi.nlm.nih.gov/) (accessed 21 June 2022 and 17 August 2022, respectively). The thresholds used in the RDP search were as follows: both type and nontype strains, both uncultured and isolates, only good sequences of >1,200 bases. For BLAST, the standard nucleotide collection database and the highly similar sequences option were used.

### Statistical analyses.

**(i) Alpha diversity (bacterial community complexity within each sample group).** Alpha diversity analysis was performed in QIIME 2 using the QIIME diversity alpha-rarefaction command and sampling depths of 50,000 sequences and 10,000 sequences. The diversity measure for 10,000 sequences was used in further statistical analysis. Three metrics were used, observed features (calculates the number of distinct sOTUs: high richness equals more sOTUs/high species diversity), Shannon index (calculates Shannon entropy of counts: diversity increases as richness and evenness increase), and faith_pd (compute Faith’s phylogenetic diversity metric: high richness equals more phylogenetically dissimilar sOTUs), taking the average after 10,000 rarefactions of 10 iterations (the number of rarefied feature tables to compute at each step). A linear mixed model was applied to test differences in alpha diversity across sample types and countries.

**(ii) Beta diversity (bacterial diversity between sample groups).** Beta diversity analysis was performed in QIIME 2 using the QIIME diversity core-metrics-phylogenetic command and a sampling depth of 10,000 sequences. The results for the different metrics (weighted and unweighted UniFrac, Bray-Curtis, and Jaccard) were visually inspected using principal-coordinate analysis (PCoA) plots.

**(iii) Filtering and data transformation.** Analyses were performed separately for sOTUs and L6 (genus level) data. The same filtration criteria were used for both L6 and sOTU data, that is, including genera/sOTUs with an average relative abundance higher than 1% or exceeding 5% in at least one sample. At L6, 68 genera were included in the analysis, whereas 151 sOTUs passed the criteria. For both tables a central log transform (CLR) (56) was applied on the relative abundances to account for the compositional nature of the data (57).

**(iv) Statistical modeling of alpha diversity and core microbiota.** Differences between sample types and countries were assessed using both univariate and multivariate analyses. A univariate linear mixed model was fitted for alpha diversity and genera identified as core microbiota using country, sample type, and household within country as explanatory variables. Country and sample type were included as fixed factors, whereas the household effect was considered a random factor and was included to account for the fact that different observations from the same kitchen may not be independent. Terms were considered significant for *P* values below 0.05. The models were fitted using lmer (from the R package lme4) (58). Different definitions are used in the literature to define a core microbiota. In this study we use the term “core microbiota” for data derived from the partial 16S rRNA gene in bacteria and define the core based on relative abundance and occurrence.

**(v) Multivariate analyses.** Principal-component analysis (PCA) and hierarchical clustering were performed for CLR transformed data (L6, after filtering). Clustering was done for both samples and genera and was visualized as heatmaps using the function pheatmap (59), for both individual samples and averaged across sample type and country (59). Euclidean distances and complete linkage were applied for the clustering of both rows (genera) and columns (averages across sample type and country); in addition, the columns were scaled.

Permutational multivariate analysis of variance (PERMANOVA) (60) and 50-50 multivariate analysis of variance (manova; ffmanova) (61) were applied to test differences in microbiota composition between countries for each of the sample types. Whereas PERMANOVA is based on distances between samples, ffmanova uses the (transformed) data as input and can, in combination with rotation tests (62, 63), identify the significantly different taxa. Adonis2 (R package vegan [62]) and ffmanova (R package ffmanova [63]) were applied for the two methods, respectively. Multivariate analyses are useful complements to the univariate models for a more holistic comparison of the microbiota. We report for which sample types the genera are significantly different between countries (false-discovery rate [FDR], <0.05) and the percentage of variation accounted for by the country effect. Only combinations of country and sample point with at least 5 observations were included in the analyses.

### Data availability.

The metagenomic sequencing data have been deposited in the National Center for Biotechnology Information (NCBI) archives under BioProject no. PRJNA878661.
