# Supplementary material for: Mapping the Kitchen Microbiota in Five European Countries Reveals a Set of Core Bacteria across Countries, Kitchen Surfaces, and Cleaning Utensils
Source: Appl Environ Microbiol. 2023 May 31;89(6):e00267-23. doi: 10.1128/aem.00267-23 (PMC10304973; doi:10.1128/aem.00267-23)
Supplement: Supplemental file 3 — Tables S1 to S3 and Fig. S1 to S3. Download aem.00267-23-s0001.docx, DOCX file, 2.3 MB [file aem.00267-23-s0001.docx]

Supplemental Material

Mapping the kitchen microbiota in five European countries reveals a set of core bacteria across countries, kitchen surfaces and cleaning utensils

Birgitte Moen, Solveig Langsrud, Ingunn Berget, Tove Maugesten and Trond Møretrø

Contents

[Supplemental tables 2](#_Toc133501842)

[***Table S1: Explained variance for country effect when running FFmanova and permanova per sample type****.* 2](#_Toc133501843)

[***Table S2. Taxa represented in the Venn diagram (L6).*** 2](#_Toc133501844)

[***Table S3. Taxa represented in the Venn diagram (sOTU) and top 10 according to mean relative abundance.*** 4](#_Toc133501845)

[Supplemental figures 6](#_Toc133501846)

[***Figure S1. Bacterial diversity between sample groups.*** 6](#_Toc133501847)

[***Figure S2. Heatmaps of the bacterial genera with average relative abundance higher than 1% or exceeding 5% in at least one sample (N=68 genera) for all samples.*** 7](#_Toc133501848)

[***Figure S3. Venn diagram with F1 (mean > 0.01), F2 (max > 0.05) and F3 present in at least one sample for all consumers.*** 8](#_Toc133501849)

[Supplemental files 8](#_Toc133501850)

[***Supplemental file S1. Relative abundance L6-table.*** *Excel file of the relative abundance at genus level (constructed from biom file) including metadata information.* 8](#_Toc133501851)

[***Supplemental file S2. Relative abundance and relative occurrence of the sOTUs.*** *Excel file of the relative abundance and occurrence of the sOTUs. The columns include: shortId, Feature.ID (sOTUs), Taxon, Confidence, class, order, family, genus and species, as well as the mean relative abundance (meanRelab) and max relative abundance (maxRelab) of the different sOTUs. In addition, the relative occurrence of each sOTU is given for samples, country and consumer, as well as for each country: France, Hungary, Norway, Portugal and Romania. Columns are further explained in the tab “Info” of the file.* 8](#_Toc133501852)

# Supplemental tables

**Table S1: Explained variance for country effect when running FFmanova and permanova per sample type**. Only countries with at least 5 samples included in the analyses. For all sample points the p-value for differences between countries is <0.001

**FFmanova**

| Sample type | ExplVar (%) | df | Countries included |
| --- | --- | --- | --- |
| Sponge | 12.040 | 2 | France,Portugal,Romania |
| Cloth | 20.087 | 2 | Norway,Portugal,Romania |
| Sink | 21.760 | 3 | France,Norway,Portugal,Romania |
| CuttingBoard | 20.756 | 3 | France,Hungary,Portugal,Romania |
| TapHandle | 21.745 | 4 | France,Hungary,Norway,Portugal,Romania |
| CounterTop | 23.073 | 4 | France,Hungary,Norway,Portugal,Romania |
| Handles | 25.552 | 4 | France,Hungary,Norway,Portugal,Romania |

**Permanova**

| Sample type | ExplVar (%)  (=partial R2*100) | df | countries |
| --- | --- | --- | --- |
| Sponge | 13.965 | 2 | France,Portugal,Romania |
| Cloth | 20.539 | 2 | Norway,Portugal,Romania |
| Sink | 21.307 | 3 | France,Norway,Portugal,Romania |
| CuttingBoard | 20.157 | 3 | France,Hungary,Portugal,Romania |
| TapHandle | 21.565 | 4 | France,Hungary,Norway,Portugal,Romania |
| CounterTop | 23.896 | 4 | France,Hungary,Norway,Portugal,Romania |
| Handles | 25.320 | 4 | France,Hungary,Norway,Portugal,Romania |

**Table S2. Taxa represented in the Venn diagram (L6).** F1= Mean: arithmetic mean abundance above a threshold of 1%; F2=MeanMax: arithmetic mean abundance above a threshold of 1% or max abundance above 5%; F3=Occurrence: taxa present in all consumers in at least one sample. MeanRelab (%)= mean relative abundance (%); Samples (%)= proportion (%) of samples where the taxa is present.

**Taxa passing all three criteria (F1+F2+F3)**

| taxa | MeanRelab (%) | Samples (%) |
| --- | --- | --- |
| *g__Acinetobacter* | 0.34 | 1.00 |
| *g__Pseudomonas* | 0.11 | 1.00 |
| *g__Enhydrobacter* | 0.07 | 1.00 |
| *f__Enterobacteriaceae* | 0.07 | 0.99 |
| *g__Psychrobacter* | 0.05 | 0.98 |
| *g__Chryseobacterium* | 0.03 | 0.99 |
| *g__Bacillus* | 0.02 | 0.89 |
| *g__Staphylococcus* | 0.02 | 0.90 |

**Taxa passing all criteria F1 and F2 (not F3)**

| taxa | MeanRelab (%) | Samples (%) |
| --- | --- | --- |
| *f__Yersiniaceae* | 0.02 | 0.85 |
| *g__Kocuria* | 0.02 | 0.88 |
| *g__Pantoea* | 0.01 | 0.71 |
| *g__Streptococcus* | 0.01 | 0.86 |

**Taxa passing all criteria F2 and F3 (not F1)**

| taxa | MeanRelab (%) | Samples (%) |
| --- | --- | --- |
| *g__Aeromonas* | 0.01 | 0.88 |
| *f__Comamonadaceae* | 0.01 | 0.97 |
| *g__Allorhizobium-Neorhizobium-Pararhizobium-Rhizobium* | 0.01 | 0.89 |

**Taxa passing only criteria F2**

| taxa | MeanRelab (%) | Samples (%) |
| --- | --- | --- |
| *g__Brevundimonas* | 0.01 | 0.93 |
| *g__Lactobacillus* | 0.01 | 0.73 |
| *g__Photobacterium* | 0.01 | 0.78 |
| *g__Stenotrophomonas* | 0.01 | 0.96 |
| *g__Lactococcus* | 0.01 | 0.91 |
| *g__Sphingomonas* | 0.01 | 0.88 |
| *g__Paracoccus* | 0.01 | 0.90 |
| *g__Micrococcus* | 0.01 | 0.72 |
| *g__Aerococcus* | 0.01 | 0.71 |
| *g__Weissella* | 0.01 | 0.43 |
| *g__Corynebacterium* | 0.00 | 0.75 |
| *o__Enterobacterales* | 0.00 | 0.27 |
| *g__Halomonas* | 0.00 | 0.25 |
| *g__Brochothrix* | 0.00 | 0.80 |
| *g__Roseomonas* | 0.00 | 0.74 |
| *g__Leuconostoc* | 0.00 | 0.75 |
| *g__Lysobacter* | 0.00 | 0.22 |
| *g__Sphingobium* | 0.00 | 0.81 |
| *g__Brevibacterium* | 0.00 | 0.63 |
| *g__Exiguobacterium* | 0.00 | 0.58 |
| *g__Vibrio* | 0.00 | 0.39 |
| *g__Sphingobacterium* | 0.00 | 0.81 |
| *g__Empedobacter* | 0.00 | 0.62 |
| *g__Massilia* | 0.00 | 0.82 |
| *g__Marinomonas* | 0.00 | 0.15 |
| *g__Methylobacterium-Methylorubrum* | 0.00 | 0.66 |
| *g__Blastocatella* | 0.00 | 0.14 |
| *g__Rothia* | 0.00 | 0.57 |
| *g__Macrococcus* | 0.00 | 0.27 |
| *g__Shewanella* | 0.00 | 0.75 |
| *g__Comamonas* | 0.00 | 0.45 |
| *g__Haemophilus* | 0.00 | 0.49 |
| *f__Sphingomonadaceae* | 0.00 | 0.44 |
| *g__Delftia* | 0.00 | 0.42 |
| *g__Flavobacterium* | 0.00 | 0.67 |
| *g__Alkanindiges* | 0.00 | 0.61 |
| *f__Caloramatoraceae* | 0.00 | 0.22 |
| *f__Micrococcaceae* | 0.00 | 0.58 |
| *g__Granulicatella* | 0.00 | 0.40 |
| *g__Duganella* | 0.00 | 0.63 |
| *g__Luteimonas* | 0.00 | 0.32 |
| *g__Carnobacterium* | 0.00 | 0.64 |
| *g__Deinococcus* | 0.00 | 0.38 |
| *g__Pseudoalteromonas* | 0.00 | 0.19 |
| *g__Psychromonas* | 0.00 | 0.13 |
| *g__Cloacibacterium* | 0.00 | 0.23 |
| *g__Rhodococcus* | 0.00 | 0.38 |
| *g__Qipengyuania* | 0.00 | 0.21 |
| *g__Escherichia-Shigella* | 0.00 | 0.14 |
| *g__Arsenicicoccus* | 0.00 | 0.04 |
| *g__uncultured* | 0.00 | 0.06 |
| *g__Paenalcaligenes* | 0.00 | 0.06 |
| *g__uncultured* | 0.00 | 0.01 |

**Table S3. Taxa represented in the Venn diagram (sOTU) and top 10 according to mean relative abundance.** F1= Mean: arithmetic mean abundance above a threshold of 1%; F2=MeanMax: arithmetic mean abundance above a threshold of 1% or max abundance above 5%; F3=Occurrence: taxa present in all consumers in at least one sample. Shortid= feature (sOTUid) corresponding to Supplementary file SX; taxa= assigned genus/family; MeanRelab (%)= mean relative abundance (%); Samples (%)= proportion (%) of samples where the taxa is present.

**Taxa passing all three criteria**

| shortid | taxa | MeanRelab (%) | Samples (%) |
| --- | --- | --- | --- |
| FT909 | *g__Enhydrobacter* | 0.07 | 1 |
| FT783 | *f__Enterobacteriaceae* | 0.04 | 0.83 |
| FT1136 | *g__Pseudomonas* | 0.04 | 0.9 |

**Taxa passing criteria F1 (mean abundance) and F2 (mean abundance and max) (not F3)**

| shortid | taxa | MeanRelab (%) | Samples (%) |
| --- | --- | --- | --- |
| FT1886 | *g__Acinetobacter* | 0.12 | 0.9 |
| FT1677 | *g__Acinetobacter* | 0.1 | 0.91 |
| FT909 | *g__Enhydrobacter* | 0.07 | 1 |
| FT783 | *f__Enterobacteriaceae* | 0.04 | 0.83 |
| FT1136 | *g__Pseudomonas* | 0.04 | 0.9 |
| FT1159 | *g__Acinetobacter* | 0.03 | 0.73 |
| FT2907 | *s__Psychrobacter_pulmonis* | 0.03 | 0.77 |
| FT714 | *g__Acinetobacter* | 0.03 | 0.52 |
| FT145 | *g__Bacillus* | 0.02 | 0.8 |
| FT1260 | *g__Acinetobacter* | 0.02 | 0.69 |
| FT2214 | *g__Chryseobacterium* | 0.02 | 0.9 |
| FT203 | *g__Pseudomonas* | 0.02 | 0.92 |
| FT771 | *g__Pseudomonas* | 0.02 | 0.79 |
| FT1183 | *f__Yersiniaceae* | 0.01 | 0.76 |
| FT2985 | *g__Staphylococcus* | 0.01 | 0.8 |
| FT1887 | *f__Enterobacteriaceae* | 0.01 | 0.39 |
| FT2643 | *g__Pantoea* | 0.01 | 0.62 |
| FT2454 | *g__Acinetobacter* | 0.01 | 0.64 |

**Top10 taxa according to mean relative abundance (sOTU)**

| shortid | taxa | MeanRelab (%) | Samples (%) |
| --- | --- | --- | --- |
| FT1886 | *g__Acinetobacter* | 0.12 | 0.9 |
| FT1677 | *g__Acinetobacter* | 0.1 | 0.91 |
| FT909 | *g__Enhydrobacter* | 0.07 | 1 |
| FT783 | *f__Enterobacteriaceae* | 0.04 | 0.83 |
| FT1136 | *g__Pseudomonas* | 0.04 | 0.9 |
| FT1159 | *g__Acinetobacter* | 0.03 | 0.73 |
| FT2907 | *s__Psychrobacter_pulmonis* | 0.03 | 0.77 |
| FT714 | *g__Acinetobacter* | 0.03 | 0.52 |
| FT145 | *g__Bacillus* | 0.02 | 0.8 |
| FT1260 | *g__Acinetobacter* | 0.02 | 0.69 |

# Supplemental figures


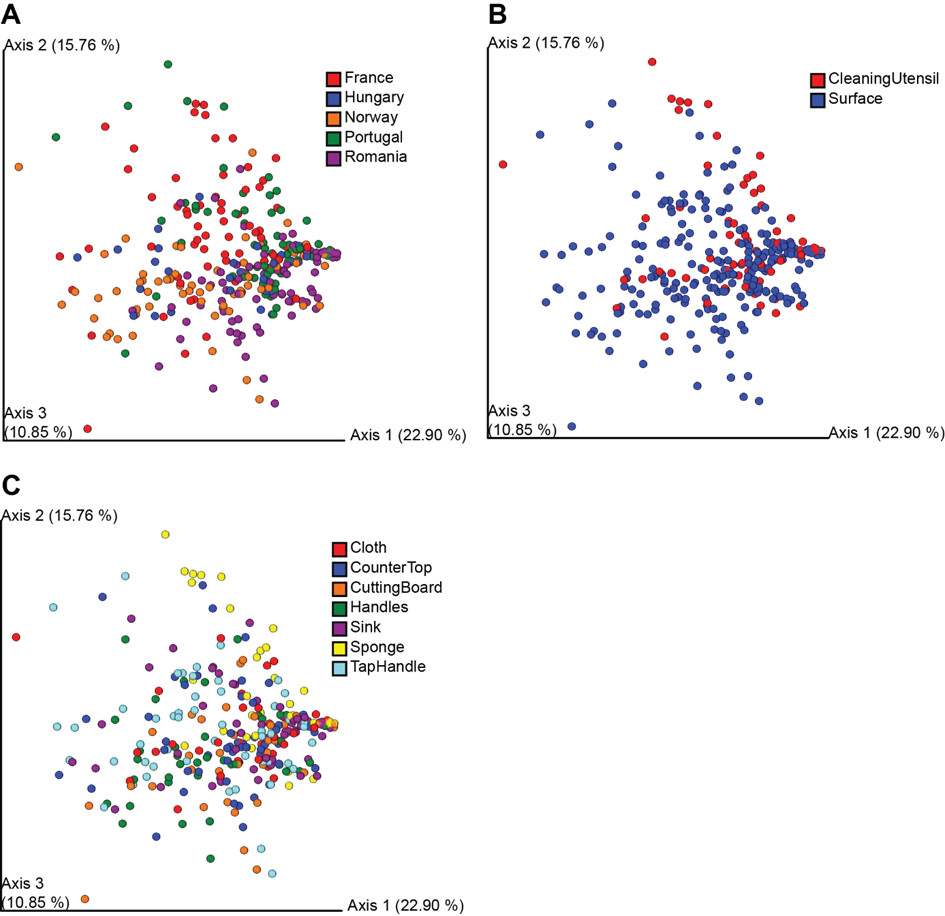


***Figure S1. Bacterial diversity between sample groups.***  *Beta diversity of all samples (rarefied to 10,000 sequences) illustrated by principal coordinates analysis (PCoA) plots based on weighted UniFrac distance. The samples are coloured based on; A: country, B: sample category (cleaning utensils and surface samples) and C: sample types.*

**
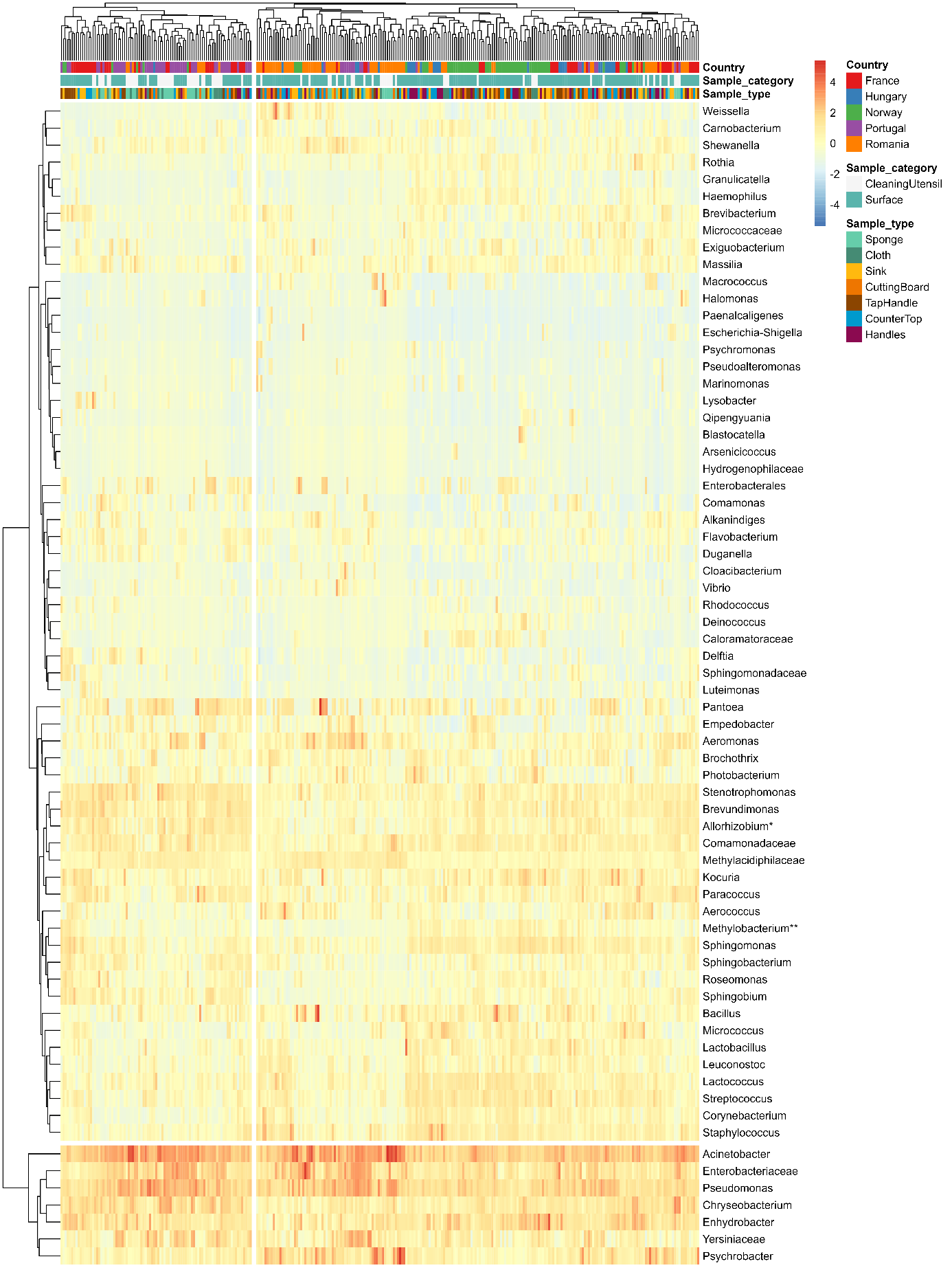
**

**Figure S2. Heatmaps of the bacterial genera with average relative abundance higher than 1% or exceeding 5% in at least one sample (N=68 genera) for all samples.** Allorhizobium* = Allorhizobium-Neorhizobium-Pararhizobium-Rhizobium, Methylobacterium** = Methylobacterium-Methylorubrum. Clustering is performed both along columns (sample type x country averages) and rows (L6 genera). The following genera are simplified Allorhizobium-Neorhizobium-Pararhizobium-Rhizobium = Allorhizobium*, and Methylobacterium-Methylorubrum = Methylobacterium**

**
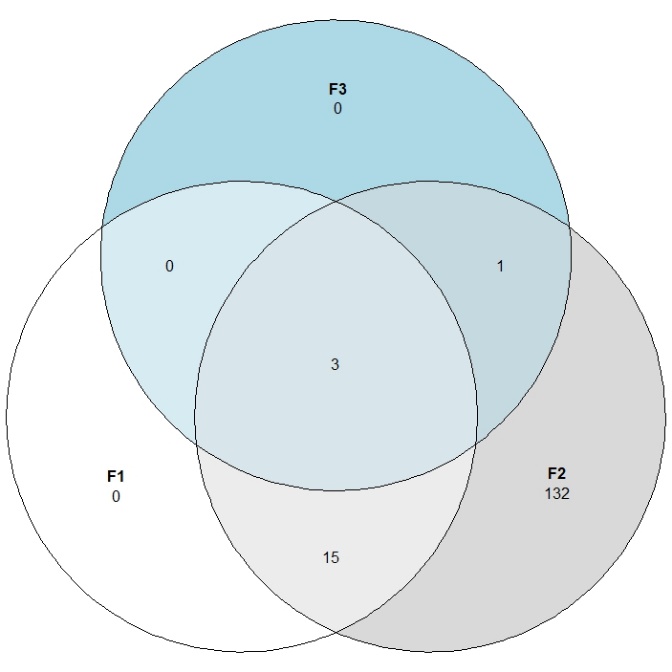
**

**Figure S3. Venn diagram with F1 (mean > 0.01), F2 (max > 0.05) and F3 present in at least one sample for all consumers.** sOTU level (feature table)

# Supplemental files

## ***Supplemental file S1. Relative abundance L6-table.*** *Excel file of the relative abundance at genus level (constructed from biom file) including metadata information.*

## ***Supplemental file S2. Relative abundance and relative occurrence of the sOTUs.*** *Excel file of the relative abundance and occurrence of the sOTUs. The columns include: shortId, Feature.ID (sOTUs), Taxon, Confidence, class, order, family, genus and species, as well as the mean relative abundance (meanRelab) and max relative abundance (maxRelab) of the different sOTUs. In addition, the relative occurrence of each sOTU is given for samples, country and consumer, as well as for each country: France, Hungary, Norway, Portugal and Romania. Columns are further explained in the tab “Info” of the file.*
